# ICU Nurses’ Knowledge, Attitude, and Practice Towards their Role in the Organ Donation Process from Brain-Dead Patients and Factors Influencing it in Iran

**Published:** 2015-08-01

**Authors:** S. T. Masoumian Hoseini, Z. Manzari, I. Khaleghi

**Affiliations:** 1 Department of Medical Surgical, School of Nursing and Midwifery, Mashhad University of Medical Science, Mashhad, Iran.; 2 Assistant Professor, Department of Medical Surgical, School of Nursing and Midwifery, Mashhad University of Medical Sciences, Mashhad, Iran; 3 Organ Procurement Unit of Mashhad University of Medical Sciences, Mashhad, Iran

**Keywords:** Knowledge, Attitude, Practice, Nurse's role, Organ donation process, Brain death

## Abstract

**Background:**

Nowadays, ICU nurses play a significant role in the care of brain-dead patients and their families. Therefore, their knowledge, attitude and practice towards this issue are extremely important to the success of organ donation.

**Objective:**

To assess ICU nurses’ knowledge, attitude and practice towards their role in the organ donation process from brain-dead patients and factors influencing it in Iran.

**Methods:**

In a cross-sectional analytical study, 90 ICU nurses working in Ghaem and Emam Reza Hospitals affiliated to Mashhad University of Medical Sciences were selected through a stratified random sampling. Data were collected from the participants by a questionnaire included demographic information, and factors influencing the nurses knowledge, attitude, and practice towards their roles in the organ donation process.

**Results:**

90 nurses participated in this study. 70% of the research subjects had spoken with their own families about organ donation; 20% had organ donation cards. The mean±SD score of nurses’ knowledge was 49.13±9.6, attitude 21.49±14.32, and practice was 3.66±6.04. 80% of nurses had a mean knowledge about their roles in the organ donation process; 82% agreed with their roles in this process, and 97% showed weak practice in this regard.

**Conclusion:**

Nurses did not have adequate knowledge, attitude, and practice towards their role in organ donation process. It is suggested to include nursing courses on the organ donation process and organ transplantation as well as educational programs to acquaint nurses with their roles in the organ donation process.

## INTRODUCTION

Following the development of science and technology and the increasing incidence of end-stage failure of vital organs such as the kidney, liver, heart, and lungs, the need for organ transplantation is rapidly increasing [1], so that the need for organ donation has increased 200% over the past two decades [2]. According to the Ministry of Health, the average statistics of brain death in Iran is 5000–6000 people a year. Furthermore, 16% of the deaths that occur in the ICU and 1%–4% of hospital deaths are primarily associated with brain death. Iran has the highest rate of brain death mortality compared to other countries [3]. Brain-dead patients worldwide are considered potential organ donors, and after the Organ Donation Act of 1999 in Iran, organ donation statistics rose from 1.7 out of a million people in 2008 to 5.7 out a million in 2011. While this figure shows only a slight increase, it reflects a growing trend of organ donation in Iran [4]. 

Numerous studies have documented evidence of the significant role of nurses, new and transparent rules and regulations, and requirements of the communities. The rate of increasing organ donation throughout the world and in our country has expanded the role of nurses [5-11], so that nowadays, the focus of nursing has shifted from special care nursing to nursing in the organ donation process [5]. According to the results of studies conducted by Polletier (1998), Coyle (2000), and Sque (2000), involvement in the organ donation process is the most positive and valuable part of the nursing profession, and nurses play a vital role in this process [6, 10, 12]. Sque (2000), and Coyle’s (2000) study showed that brain-dead patients’ families considered nurses the most effective contributor of the health care team in providing emotional support during the organ donation process [10, 12]. In addition, Watkinson (2006), Kim (2006), and Collins’ (2005) studies showed that nurses play several roles such as identifying potential organ donors, effectively following-up on the needs of brain-dead patients’ families, public education, and nursing of patients with multiple organ donors [13-15]. 

In Iran, based on the model of nursing in the organ donation process (dynamism and continuous improvement in seeking assurance and getting approve nursing model) in the Organ Transplant Center of Mashhad University of Medical Sciences, two important contributing roles and nurse advocacy in the donation process are discussed [16]. The role of nurse contribution refers to nurses informing the family of brain death and asking for organ donations as well as their role as coordinators in the process of organ donation [16]. The results of Garland’s (2010) study showed that although doctors are accountable for diagnosis and prognosis, nurses are best for reiterating the doctors’ points; therefore nurses should be present when doctors give information to the patients’ families [17]. If nurses can manage to appropriately request organ donations when they inform families of brain death, the families can more easily accept their patients’ condition and are more likely to consent to donating [8]. Nurses can act as coordinators between the treatment team, requesting team, patients and their families, and organ recipients [12, 16, 17]. Another role of the nurse is to be the patient’s advocate in the organ donation process, which plays a role in the two areas of support and protection. Support includes two components related to “patient care” and “family care.” One of the most important factors in gaining the trust of families seems to be nurse advocacy in the organ donation process, and this can be achieved by witnessing adequate patient care. Before nurse intervention, the care and treatment team should establish family trust that they will do all they can to ensure the safety and improvement of their patient, and their patient will receive enough care and treatment. The nurse’s role also includes informational support (counseling), emotional support, and bereavement support [16]. 

Despite the brief description above about nurses’ roles and their importance in the organ donation process, various studies show that ICU staff members, such as doctors and nurses, are not prepared to successfully manage the process of organ donation [18, 19]. The results of a study by Lin, *et al*, showed that the ability of nurses to spend time with families, answer their questions, and keep a positive attitude towards organ donation during the organ donation process is vital and required [11]. Mattan (1999) and Sque’s (2000) studies emphasized the important role of nurses in this process and suggested that its success is closely linked to their knowledge and beliefs towards organ donation and transplantation [12, 18]. Likewise, Keenan (2002) and Ingram’s (2002) studies indicated that the attitude of ICU nurses and doctors towards organ donation correlates to its success and indirectly increases consent [19, 20]. In the meantime, Al-Mousawi (2001) and Melo’s (2011) study accentuated that attitude and understanding of nursing towards brain death, organ donation and transplantation, and their tendency to get satisfaction of the families of patients with brain death are effective on organ donation process [21, 22].

Whither ward, the result of studies showed that having correct knowledge of organ donation process increases nurse’s self-confidence and they can answer questions of the bereaved families [14]. However, shortage of knowledge among nurses is observed practically such as making mistake in diagnosing the difference between brain death and vegetable death. Some nurses believe that patients with brain death may return to consciousness in the future. The important question is that if nurses of ICU ward do not believe in brain death as certain death, how can they have relationship with the bereaved family of organ donator and how can they talk to them [23]?

Finally, according to the aforementioned studies and a comparison between studies conducted both domestically and abroad, it is important to consider the role of nurses in the organ donation process. Studies show that nowadays, ICU nurses play a significant role in the care of brain-dead patients and their families; therefore, their knowledge, attitude and practice towards this issue are extremely important to the success of organ donation. Whither ward, in medical and nursing literature, there are a lot of articles relevant to the knowledge and attitude of health professionals. Most of these articles are about the physicians and medical students. However, we could not find any study about the knowledge, attitude, and practice of nurses towards their role in organ donation process in international medical literature. Therefore, we conducted this study to assess ICU nurses’ knowledge, attitude, and practice in relation to their role in the organ donation process from brain-dead patients and factors influencing it in Iran.

## MATERIALS AND METHODS

This cross-sectional analytical study was conducted in 2014 on ICU nurses working in Ghaem and Imam Reza Hospitals affiliated to Mashhad University of Medical Sciences, Mashhad, northeastern Iran. The minimum sample size was calculated based on the results of a pilot study; 90 nurses were selected using a stratified random sampling. The inclusion criteria included having at least one encounter with a brain-dead patient, no previous training in this field, and interest in participating in the study. Exclusion criteria included withdrawal from the study and failure to complete the study. 

This study was a student thesis, and approval was obtained from the Ethics Committee of Mashhad University of Medical Sciences. Informed consent from subjects and the right to withdraw from the study at any time were considered. Data collection tools included a questionnaire on demographic information, a questionnaire on factors influencing nurse’s knowledge, attitude, and practice during the organ donation process, and a questionnaire surveying “nurse’s knowledge, attitude and practice in relation to their roles in the organ donation process.” The nine-question form studied factors influencing nurses’ knowledge, attitude and practice in the organ donation process along with questions about having an organ donation card, caring for brain-dead patients, speaking with families about organ donation, *etc*. The questionnaire “nurse’s knowledge, attitude, and practice in relation to their role in the organ donation process” was based and designed on a dynamism and continuous improvement in seeking assurance and getting approve nursing model that introduced nurse roles in the organ donation process in Iran [16]. The questionnaire consists of 29 yes/no and multiple-choice questions; scores were between zero and 29. The second part of the survey included 13 questions on a seven-point Likert scale about nurse’s attitude towards their role in the organ donation process. Participants placed their responses on a continuum for each statement from “very strongly agree” to “very strongly disagree.” Items were weighted from +3 to 3, respectively. Therefore, the possible range of score was from 39 to +39; higher scores indicate more positive attitude towards nurse’s role in organ donation process. This section of the questionnaire also consisted of three items (2, 5, 12) that were reverse scores. These items weighted from 3 “very strongly agree” to +3 “very strongly disagree.” The third section of the questionnaire that was designed to evaluate nurse’s practice in relation to their role in the organ donation process, included two factual scenarios of brain-dead patients in Shahid Kamyab Hospital in Mashhad, written by the researcher. The first scenario was related to the role of nurse’s advocacy in the organ donation process, and the second was related to nurse’s participatory roles in this process. Each scenario was followed by five essay questions (10 total). The score ranged from a minimum of zero (the lowest nurse’s practice in the organ donation process), to a maximum of 10 (the highest nurse’s practice). 

Content validity of study tools was confirmed. A test-retest method was used to test the reliability of the questionnaire of factors influencing nurse’s knowledge, attitude and practice during the organ donation process (r=0.86). A Kuder-Richardson 20 was used to evaluate the reliability of the questionnaire of nurses’ knowledge about their role in the organ donation process (r=0.88); reliability of the attitude questionnaire was found very good (Cronbach’s α of 0.96). Inter-rater agreement was used to assess the reliability of the practice questionnaire (r=0.87). The questionnaires were completed by participants in the ICU of Ghaem and Emam Reza Hospitals in the presence of the researcher. SPSS^®^ ver 11.5 for Windows^®^ was used to analyze the data. Pearson correlation coefficient was used to assess the relationship between knowledge, attitude, and practice variables. Scores for knowledge, attitude, and practice were calculated on a scale of 100. The results for knowledge and practice were divided into five categories—“very high,” “high,” “medium,” “low,” and “very low.” The results of the attitude were categorized into seven groups—“very strongly agree,” “strongly agree,” “agree,” “no opinion,” “disagree,” “strongly disagree,” “very strongly disagree.” 

Normality of the data distribution was assessed by Kolmogorov-Smirnov and Shapiro-Wilk test. Knowledge, attitude, and practice variables had normal distribution. A p value <0.05 was considered statistically significant.

## RESULTS

The study sample was confined to two large Mashhad University of Medical Sciences affiliated hospitals and that their ICU nurses have been exposed to a broad range of experience and a greater likelihood of contact with potential organ donor and their families. Ghaem Hospital with 130 nurses working in nine ICUs, and Imam Reza Hospital with 70 nurses working in four ICUs, serve patients.

Ninety nurses participated in this study. All of them answered the questionnaires; 77% of the nurses were married; 23% were single; 82% were female. The mean±SD age of participants was 33.2±5.8 (range: 24–56) years. Half of the nurses were contract-based; and 97% held a bachelor’s degree in nursing. The majority (92%) was clinical nurses; 8% were head nurses. Their mean±SD total work experience as nurses was 9.1±6.1 years; they had a mean±SD work experience of 6.0±4.7 years in ICU. Almost two-thirds (65%) of the nurses had at least one experience caring for a brain-dead patient during their career; 92% of them had at least once during their career, introduced a patient suspected brain death to the organ procurement unit; 44% were present at least once when the doctor delivered the news of brain death to the patient’s family; 70% had spoken with their own families about organ donation; and 20% had organ donation cards. 

The mean±SD score of nurses’ knowledge was 49.13±9.6 (range: 24.14–79.31). Nurses› attitude had a mean±SD score of 21.49±14.32 (range: 35.90–37.46). It was 3.66±6.04 (range: 0–22.50) for practice score. Eighty percent of nurses had an average knowledge about their role in the organ donation process; 82% agreed upon their role in the process; and 97% had weak practice in this regard. 

The mean nurses’ knowledge score was significantly (p=0.04) directly correlated with their work experience in the ICU. Nurses who were head nurses were more knowledgeable about their roles in the organ donation process. There were also significant direct correlation between the practice score and work experience in ICU (p<0.001) and also total work experience as a nurse (p<0.003). Nurse’s attitude score and total work experience as a nurse also had a significant (p<0.001) direct correlation. Age and knowledge also had a significant (p<0.02) correlation so that older nurses were more knowledgeable in this area. However, no significant correlation was observed between attitude and work experience in the ICU (p=0.14). Knowledge was not associated with sex (p=0.65) and marital status (p=0.62). Attitude was neither associated with sex (p=0.67) nor marital status (p=0.60). There was no significant correlation between practice and gender (p=0.98) or marital status (p=0.14). There were significant direct correlations between nurse’s knowledge and practice (p<0.03), and nurse’s attitude and practice (p<0.02). However, nurse’s knowledge and attitude did not have any significant correlation (p=0.28).

It was found that those nurses who had spoken to families about organ donations showed a significantly higher attitude (p<0.002) and practice (p<0.001) in these areas than those who had not. Likewise, those nurses who had organ donation card had higher knowledge (p<0.02), attitude (p<0.01), and practice (p<0.04) compared to those who did not have (Table 1). A significant differences was also observed between nurses who had an organ donation card and those who had spoken to their families about organ donations (p<0.01). There was no significant differences between nurses who were present when doctors delivered news about brain-dead patients and nurse’s knowledge (p=0.54), attitude (p=0.53), and practice (p=0.73). There was also no significant differences between referring a brain-dead patient to the organ procurement unit and nurse’s knowledge (p=0.64), attitude (p=0.19), and practice (p=0.30) in relation to nurse’s role in organ donation process. Likewise, no significant differences was observed between caring for a brain-dead patient and knowledge (p=0.90), attitude (p=0.86), and practice (p=0.52) of studied nurses during the organ donation process. There was no statistically significant difference between having an organ donation card and demographic characteristics of the study group. None of the participants had previously received training in this field. There was no history of being an organ donor or recipient among their family members. 

**Table 1:** Mean±SD nurses’ knowledge, attitude and practice scores stratified by having organ donor card

Variables	Organ Donor Card		p value
	Yes	No	
Knowledge	54.5±10.5	47.7±8.9	0.02
Attitude	28.0±7.7	19.9±15.1	0.01
Practice	7.9±8.9	2.6±4.6	0.04

## DISCUSSION

This study was conducted with the aim of studying knowledge, attitude, and practice of ICU nurses towards their roles in the organ donation process of brain-dead patients in Iran. It is one of the first studies conducted in Iran and worldwide in this regard. The general situation of nurses in this study in relation to their roles in the organ donation process of brain-dead patients showed that 80% of them had an average knowledge. Likewise, 81.7% of nurses agreed with their roles in the organ donation process. The practice results showed that 96.7% of nurses had a weak self-practice. These results matched the results of a study by Bidigare and Oermann (1991) that showed inadequate nurses’ knowledge about organ procurement process, identifying potential organ donors, and brain death as well as its criteria [24]. The results of many studies indicated that generally, doctors and nurses do not have enough knowledge about the organ donation process of brain-dead patients. Naserollazadeh, *et al*, studied factors influencing knowledge and attitude of nurses working in the kidney transplant unit towards organ transplantation from cadavers. It was found that 84.6% of the participants had favorable attitudes toward donation; 67% equated brain death with death, while only 40% had a correct definition of brain death [25]. Our study showed a significant relationship between age and attitude of the participants; however, we could not find any correlation between knowledge and attitude of participants, which was inconsistent with the results of Naserollahzadeh. In Naserollahzadeh’s study, there was no significant relationship between age and attitude of participants; also, there was a direct correlation between a negative attitude and misunderstanding of the concept of brain death [25].

In the study by Rachmani, aimed at evaluating doctors and nurses’ knowledge and attitude in the field of organ transplants, it was shown that health care employees and doctors had little tendency to participate directly in this process and had little knowledge about brain death [26]. Similar to these results, our study also showed that nurses had weak knowledge, attitude, and practice towards their roles in the organ donation process. Rachmani showed that a strong direct correlation existed between the knowledge and attitudes of health care workers and doctors. The present study may differ from Rachmani’s study for the cultural context and social, health, and educational policies in Iran. It should be noted that organ transplantation courses are part of the curriculum for doctors and nurses in that country [26], while they are not in Iran [27]. Akgun, *et al*, also evaluated the knowledge and attitude of health care workers. Unlike our study, there was a direct strong correlation between knowledge and attitude of subjects. Despite acceptable results regarding doctors and nurses’ knowledge and attitude towards organ and tissue donation, there was one drawback that is the low willingness of the study population to donate their own or their relatives’ organs. In this study, it was concluded that knowledge alone was not effective for increasing organ donation participation, and opportunities and incentives should be provided to implement this practice [28]. In addition to the existence of cultural, social, economic and health policy differences in Turkey and Iran, the observed difference may be attributed to the sample size of the present study (90 participants) compared to Akgun’s study (1184 participants), because one of the factors affecting correlation is sample size.

Similar to our study, Kim, *et al*, showed a strong direct relationship between knowledge and attitude among health care professionals in relation to brain death and organ donation. Notable in this study was that even though health care professionals showed positive attitudes towards organ donation, 74.1% expressed difficulty in suggesting the option to families of brain-dead patients. The results indicated that while families were aware of the importance of organ donation, they were not accepting of the reality in practice. This might be a reflection of Korean culture, which believes that the soul remains in the body after death. The results also indicated that participation in the organ donation process affected knowledge more than attitude [29].The present study indicated that nurses’ practice in relation to their roles in the organ donation process while affected by knowledge, was also influenced by their attitude. Those who had a more positive attitude towards their roles in the organ donation process showed better practice. The results of a study by Matten, *et al*, also showed that positive opinions and nurses’ attitudes had a significant correlation with their participation in consultation about organ donation with brain-dead patients’ families and a consequent increase in donation rate. The authors therefore concluded that the success rate of organ donation was directly related to the attitude and practices of nurses [18]. 

Although 81.7% of the subjects in the present study agreed with the role of nurses in the organ donation process, only 20% of them had organ donation cards. This may be due to cultural factors and other variables in Iran because many find the subject of organ transplants from brain-dead patients inconsistent with Islam, the official religion, despite the approval of religious scholars. While many people have a positive attitude towards this issue, many do not get an organ donation card due to psychological pressure and opposition from family members. In this respect, the results of the study by Zohur, *et al*, which evaluated the attitudes of doctors and nurses in the ICU in the University of Medical Sciences in Iran towards organ donation of brain-dead patients showed that about 95% of doctors and nurses agreed with transplantation from brain-dead patients, but only 79% of them had completed and signed an organ donation card. It is noteworthy that despite the positive attitude of nurses towards organ donation, they expressed such attitude when they did not have much knowledge about brain death [30]. In a study conducted by Akgun, *et al*, although 44.2% of nurses expressed a willingness to donate their organs, only 17.9% had an organ donation card [28]. This suggests that even though nurses may express a positive attitude, they fail to realize their beliefs. Vahidi, *et al*, evaluated the health care staffs’ belief about organ donation at Tabriz University of Medical Sciences and showed that 52% of the subjects had never thought about organ donation, and two-thirds of them expressed a lack of enough confidence to suggest organ donation to the relatives of brain-dead patients because they believed this would add to their grief [31]. The results of a study by Ozdag showed that even though 87.7% of nurses in the study had a positive attitude towards organ donation, only 10.8% were aware of organ donation laws. This further indicated that nurses express positive attitudes when they have little knowledge about organ donation. Additionally, 58.7% of nurses in the study had an organ donation card [32], which was more than two times the rate of nurses with an organ donation card in our study. This difference may be due to the presence of motivational programs in the field of organ donation and transplantation as well as the accessibility in obtaining an organ donation card in social context of this study. In the present study, nurses with an organ donation card had higher knowledge, attitude, and practice compared to nurses who did not have a card, which is compatible with the results of the study by Aghayan, *et al*, who evaluated the rate of knowledge and attitude of nurses in the ICU and emergency department in the field of organ and tissue transplants. Those with an organ donation card had significantly higher knowledge and attitudes compared to others, which indicates the effectiveness of more knowledge and a positive attitude for obtaining a card. As a result, they were better accepting organ donation. Like Aghayan, *et al*’s study, our study showed a significant correlation between nurses’ work experience and their attitude, so that nurses with more work experience had a more positive attitude towards organ donation. Additionally, our study did not show a significant correlation between having the experience of introducing a brain-dead patient to organ procurement unit and nurses’ knowledge, attitude, and practice towards their roles in the organ donation process. This result was contrary to the Aghayan’s study, where nurses with more knowledge and positive attitudes attempted to introduce more potential organ donors to procurement unit [33]. The reason for the difference between these studies may be attributed to different hospital policies. In Ghaem and Emam Reza Hospitals, no protocol existed on how to deal with cases of brain death. Furthermore, we evaluated knowledge, attitude, and practice towards nurses’ roles in the organ donation process, whereas Aghayan focused on the process of organ donation. 

Limitations on the present study include use of a questionnaire to evaluate nurses’ practice in the organ donation process. There was also limited exposure to brain-dead patients in a clinical setting for each nurse in the study, and the time limits of the survey did not allow for the use of a checklist. The study sample was confined to two large Mashhad hospitals; it is possible that their nurses had been exposed to a broader range of experience and a greater likelihood of contact with brain-dead patients and their families comrade to smaller hospitals. Moreover, these were educational hospitals. Ideally the study should have been done in different hospitals in our territory.

Numerous weaknesses exist in the training of nurses both in universities and work place. In academic and clinical training, there has not been as much attention given to nurses involved in the organ donation process as there has been to transplant nurses, despite being cited in the nursing curriculum in Iran. As a result, the nurses do not have adequate knowledge, attitude, and practice. There is, however, an undeniable link in their role as influential pioneers in the improvement of this field. Therefore, it is suggested to include nursing courses in organ donation process and organ transplantation as well as educational programs to acquaint nurses with their roles in the process to improve their attitude and practice by different training methods.

## Notes

**FINANCIAL SUPPORT:** Mashhad University of Medical Sciences
